# Air, Water, Sewerage and Drainage

**Published:** 1871-02

**Authors:** 


### AIR, WATER, SEWERAGE AND DRAINAGE.

Fresh air is a necessity of life, a first condition of health; but city air is no longer the purifying, life-giving body which is met with on the sea-shore or the mountain-top. Its ozone is exhausted, it is laden with poisons from multitudinous chimneys, and a dense crowd of organic impurities are revealed by the microscope or the transient sunbeam. In the courts and alleys it does not improve; it is worse still in the crowded workshop or the crowded house, where ventillation is ignored and the laws of cubic space unknown.

Next in importance to air, and equally with it a necessity of life and a condition of health, is water. Pure air and pure water are man's right, and no change of place should make them inaccessible. Not only a wholesome supply for drinking, but baths and wash-houses should in every town be within the reach of the very poor. Dirt and disease are inseparable, and it should be a lasting disgrace to the community that renders both inevitable by a water supply which is either impure or insufficient for the wants of the population.

The source of water supply is perhaps the most momentous problem which a town's population can have to solve, and it has acquired still greater importance from recent investigations respecting the spread of disease; yet not only the source, but the quantity, distribution, and even quality of the water, are not unfrequently in the hands of those whose interests are not those of the consumers. A trading monopoly may decimate by cholera and diarrhœa, may degrade in filth and depravity, the poor of a town, which in cruel mockery, they are said to serve. On the subject of purity much difference of opinion still unhappily exists. What amount of impurity, what amount of animal pollution, if any, can be consumed with safety? Does filtration, does exposure to air in the river's course, convert a sewage-laden water into a wholesome drink? are questions still seething in the crucibles of rival chemists,—questions which it may yet be that the physiologist will be called upon to decide. Low levels will receive the drainage from higher parts and of streets as well. Sewers will leak or get blocked, old cesspools are never in repair, yet pumps are still used in the densest quarters of our towns and cities, and, as might be expected, are the fruitful source of typhoid and choleraic disease.

"Is industry free to tumble out whatever horror of refuse it may have arrived at into the nearest crystal brook, regardless of gods and men and little fishes; is free industry free to convert all our rivers into sewers?" exclaims a modern writer, and with reason; but an indignant protest is one thing, and a practical remedy for the evils declaimed against, another.

"It is ours to use air and water," says Dr. Gairdner, "and then to pass them on; but woe to the man or community that detains or imprisons these, his servants of the hour, in their further execution of God's endless work." The danger is now too well known to be commented upon, and it is not going too far to say that the disposal of sewage is one of the great sanitary questions of the day. River pollution has assumed gigantic proportions; yet the difficulties—sanitary, engineering and agricultural—in the way of change are appalling; so great indeed that they have led thinking men to go back once more to the first rudiments of sanitary science, there, haply, to find wisdom and the right way. No single system, however, can be expected to accommodate itself to the outfall, the soil, and the topographical as well as social conditions of each several centre of population."

Drainage, though closely allied to sewage, has its very distinct purposes in the economy of health; and though the latter may be the most pressing, the former is equally important in the removal of unsanitary conditions.

The drainage of a town, however, may be complete, and yet if the house drains are untrapped, or out of repair, the results are no less disastrous.

Indeed the more perfect, and the more impervious are the sewers, the greater the danger from the admission of typhoid and other poisons to our dwellings through every unguarded avenue in the drains which communicate with them. The facts bearing on this subject are innumerable, and a small pamphlet by Dr. Carpenter, of Croydon, England, called "Hints on House Drainage," may be mentioned as giving in the smallest compass such information on the subject as every householder should possess and ponder, if he values the health of his family and those under his roof.

The stench from a tallow factory or other offensive trade may pervade whole districts of a town, and sicken all within its reach. Pigsties may exist in back courts and alleys, may poison wells with impunity, and be the bane of a whole neighborhood. Slaughter-houses may remain in the very centre of the cleanest (?) towns, unnoticed, perhaps, but none the less dangerous ; and yet if no actual case of acute disease can be attributed to them, if not offensive to the eyes or nose of the inspector (appointed most frequently without other qualification than that of being right in politics) they are no nuisance in the eye of the law, and may continue unchecked in their silent work of sapping at their very foundations the health and strength of the people.—*Good Health.*

